# Cardiovascular disease prevention in rural Nigeria in the context of a community based health insurance scheme: QUality Improvement Cardiovascular care Kwara-I (QUICK-I)

**DOI:** 10.1186/1471-2458-11-186

**Published:** 2011-03-25

**Authors:** Marleen Hendriks, Lizzy Brewster, Ferdinand Wit, Oladimeji Akeem Bolarinwa, Aina Olufemi Odusola, William Redekop, Navin Bindraban, Albert Vollaard, Shade Alli, Peju Adenusi, Kayode Agbede, Tanimola Akande, Joep Lange, Constance Schultsz

**Affiliations:** 1 Dept of Global Health, Academic Medical Center, University of Amsterdam, Pietersbergweg 17, Amsterdam, 1105 BM, The Netherlands; 2 Dept of Neurology, Academic Medical Center, University of Amsterdam, Meibergdreef 9, Amsterdam, 1105 AZ, The Netherlands; 3 Depts of Internal and Vascular Medicine, Academic Medical Center, University of Amsterdam, Meibergdreef 9, Amsterdam, 1105 AZ, The Netherlands; 4 Dept of Epidemiology and Community Health, University of Ilorin Teaching Hospital, P.M.B. 1459, Ilorin, postal code 240001, Nigeria; 5 PharmAccess Foundation, 1c Raymond Njoku Street, S.W. Ikoyi, Lagos, Nigeria; 6 Institute for Medical Technology Assessment, Erasmus University Rotterdam, Burgemeester Oudlaan 50, Rotterdam, 3062 PA, The Netherlands; 7 Dept of Cardiology, Academic Medical Center, University of Amsterdam, Meibergdreef 9, Amsterdam, 1105 AZ, The Netherlands; 8 PharmAccess Foundation, Pietersbergweg 17, Amsterdam, 1105 BM, The Netherlands; 9 Dept of Cardiology, Lagoon Hospitals, 8 Marine Road, Apapa, Lagos, Nigeria; 10 Hygeia Nigeria Ltd, 13B Idejo Street, Victoria Island, Lagos, Nigeria; 11 Ogo Oluwa Hospital, 64/65 Ahmadu Bello Way, Bacita, Kwara State, Nigeria; 12 Oxford University Clinical Research Unit, Hospital for Tropical Diseases, 190 Ben Ham Tu, Ho Chi Min City, District 5, Vietnam

## Abstract

**Background:**

Cardiovascular diseases (CVD) are a leading contributor to the burden of disease in low- and middle-income countries. Guidelines for CVD prevention care in low resource settings have been developed but little information is available on strategies to implement this care. A community health insurance program might be used to improve patients' access to care. The operational research project "QUality Improvement Cardiovascular care Kwara - I (QUICK-I)" aims to assess the feasibility of CVD prevention care in rural Nigeria, according to international guidelines, in the context of a community based health insurance scheme.

**Methods/Design:**

*Design: *prospective observational hospital based cohort study.

*Setting: *a primary health care centre in rural Nigeria.

*Study population: *300 patients at risk for development of CVD (patients with hypertension, diabetes, renal disease or established CVD) who are enrolled in the Hygeia Community Health Plan.

*Measurements: *demographic and socio- economic data, physical and laboratory examination, CVD risk profile including screening for target organ damage. Measurements will be done at 3 month intervals during 1 year. Direct and indirect costs of CVD prevention care will be estimated.

*Outcomes: *1) The adjusted cardiovascular quality of care indicator scores based on the "United Kingdom National Health Services Quality and Outcome Framework". 2) The average costs of CVD prevention and treatment per patient per year for patients, the clinic and the insurance company. 3) The estimated net health care costs of standard CVD prevention care per quality-adjusted life year gained.

*Analysis: *The primary outcomes, the score on CVD quality indicators and cost data will be descriptive. The quality scores and cost data will be used to describe the feasibility of CVD prevention care according to international guidelines. A cost-effectiveness analysis will be done using a Markov model.

**Discussion:**

Results of QUICK-I can be used by policy makers and professionals who aim to implement CVD prevention programs in settings with limited resources. The context of the insurance program will provide insight in the opportunities community health insurance may offer to attain sustainable chronic disease management programs in low resource settings.

**Trial registration:**

This protocol has been registered at ISRCTN, ID number: ISRCTN47894401.

## Background

Cardiovascular diseases (CVD) are well established as a leading contributor to the burden of disease in low-income and middle-income countries (LMIC). Over 80% of global CVD mortality occurs in LMIC [1]. The burden of non-communicable diseases in LMIC is likely to increase substantially over the next decades, and will become one of the most important causes of death and disability, because of the expected health and demographic transition [2,3]. CVD in sub-Saharan Africa (SSA) has a large economic impact, with an estimated financial burden of tens of billions of US dollars over the next decade [4,5]. Interventions to prevent CVD, such as multidrug regimes for high risk individuals, are available and were found cost effective in LMIC in modelling studies [6,7] but coverage in these settings is low [8].

The need for control of non communicable diseases and implementation of CVD prevention programs in LMIC was recently emphasized [9-14]. The World Health Organization and the International Society of Hypertension (WHO/ISH) have jointly published guidelines for the treatment of CVD risk factors in LMIC [15-17]. However, implementation of CVD prevention programs poses several challenges. The most important is that health care for chronic diseases requires a greater level of organization of care that must be sustained over a longer period of time (usually a patients' lifetime) than care for acute problems. In addition, access to health care, in particular affordability of drugs and travel, can pose restraint on the prospective patients. This was demonstrated in the USA where limited access to care due to lack of insurance, was associated with increased levels of forgone health care [18]. Therefore, it was recently argued that interventions for CVD prevention in LMIC must be logistically and financially feasible and that operational research of implementation programs is urgently needed [19]. In this paper, we present an operational research project to assess the feasibility of standard CVD prevention care according to international guidelines in rural Nigeria, in the context of a subsidized, community based health insurance program.

### The insurance program: Health Insurance Fund

Fifty percent of SSA's total health expenditure is financed by out of pocket payments, causing many to fall into a poverty trap having to pay for healthcare services [20,21]. The Health Insurance Fund (HIF) is an international not-for-profit organization committed to the delivery of affordable quality private health insurance and healthcare services for low-and middle-income families. The mission of the HIF program is to protect the wealth of low- and middle- income families from health-related risks, so that families can lead lives of self-reliance, and meet their basic needs. To achieve this mission the HIF program is building a healthcare financing and delivery system that is centred around private health insurance.

### The Health Insurance Fund in Nigeria

The first two HIF programs started in early 2007 in Nigeria in Lagos and Kwara State under the name of Hygeia Community Health Plan (HCHP). The implementation of the HIF program in Nigeria is carried out by PharmAccess Foundation and the local Health Maintenance Organization Hygeia Nigeria Ltd. Hygeia has contracted both private and public clinics to provide the care for the enrolees. The HCHP program provides access to care for patients, performs upgrading of all clinics participating in the program, implements guidelines, assists the health care providers in improving financial and administrative management in the clinic and monitors and evaluates these processes. The insurance package provides coverage for the most common medical problems found among the target groups and consists of primary care, and limited secondary care (limited hospitalization and basic surgery). A more detailed description of the insurance program can be found at HIF's website [22].

Treatment for CVD risk factors such as hypertension and diabetes is included in the insurance package. Before the start of the HCHP program, guidelines for CVD prevention care were not available and very few patients were treated for CVD risk factors. As part of the HCHP program, staff was trained in CVD risk management including treatment guidelines (see Additional file 1) and other relevant topics, such as ECG reading.

### Aim of the study

**Figure 1:**
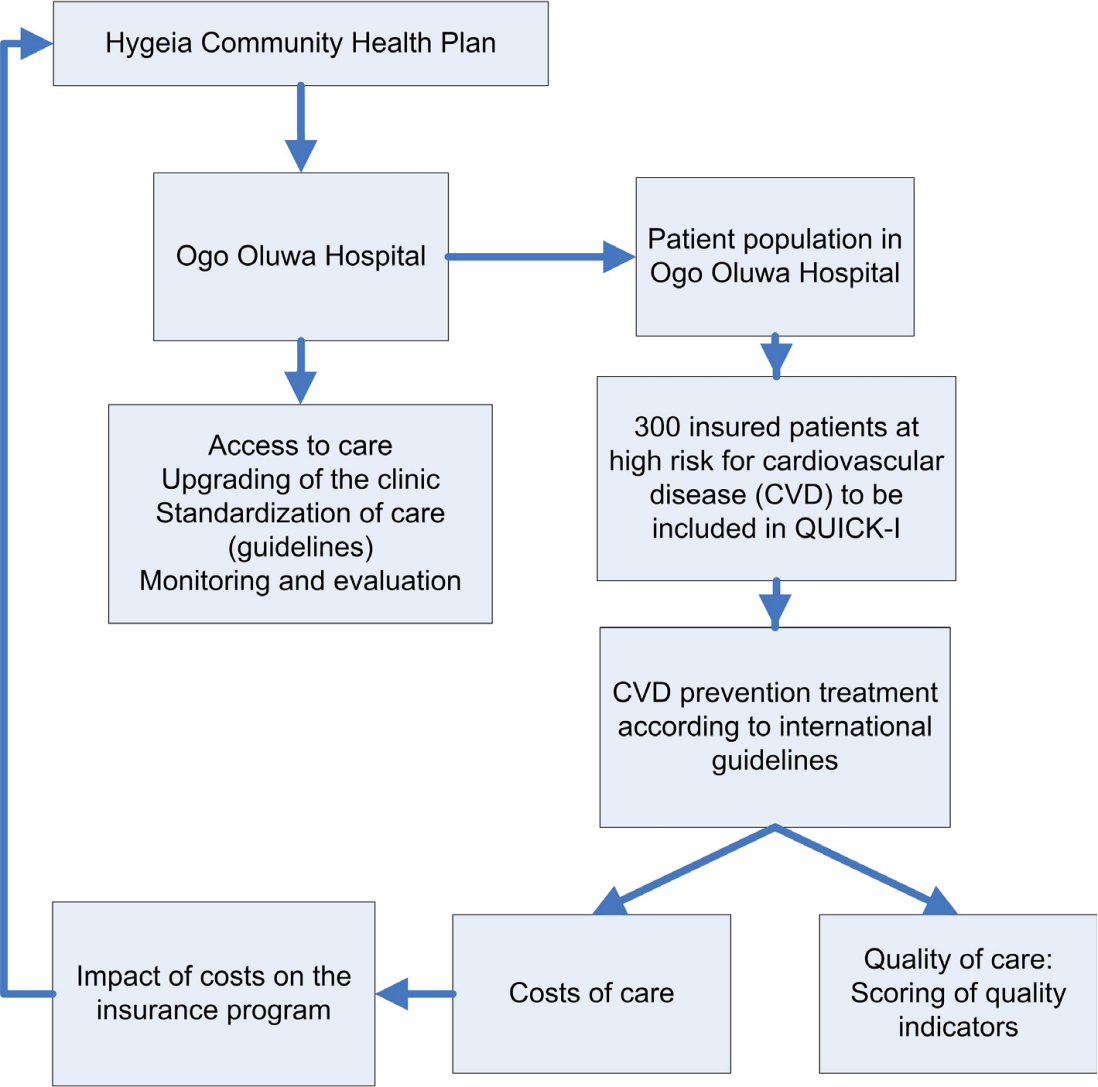
**General outline of QUality Improvement Cardiovascular care Kwara-I (QUICK-I) within the Hygeia Community Health Plan**.

It is hypothesized that the HCHP program can be used to provide high quality CVD prevention care according to international guidelines by covering the costs of the CVD prevention care (access to care). The project QUality Improvement Cardiovascular care Kwara-I (QUICK-I) aims to assess the feasibility of standard CVD prevention care according to international guidelines, in a low resource setting, in the context of the HCHP health insurance scheme. Both operational feasibility of CVD care in the clinic (e.g. availability of laboratory tests, drugs, doctors) and financial feasibility (for the insurer, the clinic and the patients) will be evaluated. Operational feasibility will be assessed by measuring the quality of CVD prevention care in the study clinic after the implementation of the CVD prevention program. Financial feasibility will be assessed by estimating the costs of the CVD prevention program. Figure 1 gives a general overview of the QUICK-I project within the HCHP program. In addition, patient-related determinants of feasibility, focused on patient education and drug adherence, will be studied simultaneously, and is described elsewhere [23].

## Methods

### Project Design

This is a prospective observational hospital based cohort study.

### Setting

#### Study clinic

The clinic participating in this project is Ogo Oluwa Hospital (OOH) in Bacita (Kwara State), a private clinic in rural Nigeria that participates in the HCHP program. The hospital has admission capability and operates very busy weekly primary care clinics for the management of CVD prevention care. At most times, at least 3 doctors, 6 nurses, 3 laboratory staff, 3 records staff, 2 pharmacy staff, 2 administrative staff and several other support staff are available during service hours. At the time of writing, 9500 patients enrolled in the HCHP program, were registered in OOH. As almost all patients consulting OOH are enrolled in the HCHP program, this number can be considered an estimate of the total patient population registered in OOH.

#### Population

OOH provides care for the people who live in the area around the community of Bacita. This population is a rural, low income, farming community, predominantly Muslim and belongs mostly to the Nupe and Yoruba ethnic groups. Bacita is 1 of the 4 most populous communities in Edu Local Government Area. Edu has an estimated population size of 201,469 [24].

### Inclusion and exclusion criteria

Table 1 shows the inclusion and exclusion criteria of the study. In order to be included a patient needs to meet all inclusion criteria and none of the exclusion criteria. Patients who do not meet the inclusion criteria or who meet one of the exclusion criteria will receive the same standard care in the clinic but will not be included in the cohort.

**Table 1:** Inclusion and exclusion criteria for QUality Improvement Cardiovascular care Kwara-I

Inclusion criteria	1. ≥ 18 years of age
	2. Visiting the outpatient clinic/admitted to the clinic
	3. Enrolled in the Hygeia Community Health Plan
	4. At least one of the following:
	a. diagnosis of hypertension
	b. diagnosis of diabetes mellitus
	c. established cardiovascular disease (stroke, myocardial infarction, angina pectoris)
	d. diagnosis of renal disease
Exclusion criteria	1. Patients who are unwilling to provide consent for data collection
	2. All pregnant or lactating females
	3. All patients with suspected secondary hypertension
	4. Any person who is incapable of giving informed consent
	5. Patients who are not permanently residing in Kwara State*

*Patients who are not permanently residing in Kwara State are not eligible for the HCHP program.

### Project Duration

The inclusion of patients in the cohort is expected to take 6 months. Each patient will be followed for 1 year. Total duration of data collection is therefore estimated to be 18 months. After the project has finished, CVD prevention care will be continued according to the implemented guidelines.

### Investigations and Follow up

Patients who are included in the project will be assessed every 12 months according to current CVD prevention guidelines [15-17,25-27]. In addition, regular follow up visits will take place with the doctors according to local practice of care (usually patients are seen by the doctors once a month). In addition, for study purposes, patients are seen every 3 months during 1 year by a dedicated nurse who assists in data collection. The data collected during each visit are shown in Table 2.

**Table 2:** Data collection and investigations during each study visit

Month	0	3	6	9	12
Demographic data	X				

Socioeconomic data	X				

Co morbidities	X				

Cardiovascular risk factors	X		X		X

Transport costs	X	X	X	X	X

Drug use for cardiovascular disease (prevention) and side effects	X	X	X	X	X

Morisky adherence questionnaire [40]	X	X	X	X	X

Rose angina pectoris questionnaire [41,42]	X				

Quality of life using the 12-Item Short Form Health Survey (SF-12) [43]	X				X

Health care utilization and health care expenditures		X	X	X	X

Cardiovascular events		X	X	X	X

Physical Examination (height, weight, hip and waist circumference, blood pressure, heart rate)	X	X	X	X	X

Blood tests: (potassium, creatinine, lipid profile)	X				X

Blood tests: glucose	X	X*	X*	X*	X

Urine tests (microalbuminuria, proteinuria**)	X				X

Electrocardiogram	X				X

*Only for diabetic patients

** Only if microalbuminuria test result is out of range at upper measuring range

Patients will be reminded of their upcoming study visits using telephone calls or text messages. Patients, who do not show up for study visits, will be contacted (if needed by home visit) by a study staff member to investigate the reasons for no show. Rates of loss to follow up and reasons for no show will be described as part of the feasibility of CVD prevention care.

### Primary Endpoints

The primary endpoints of QUICK-I are:

A. The adjusted cardiovascular care quality scores based on the United Kingdom National Health Services Quality and Outcome framework (NHS QOF) [28] after the implementation of the CVD prevention program. The score will be measured after the last patient has completed follow up (estimated 1.5 years after baseline). The quality indicators are shown in Additional file 2.

B. The range of possible costs of CVD prevention treatment per patient per year, divided per category (treatment, consultation etc). Pre-specified subgroup analysis will include costs for the insurance company considering the current and alternative benefit packages, the hospital, and the patient.

C. The estimated net health care costs of standard CVD prevention care per quality-adjusted life year (QALY) gained in a community health insurance setting.

### Secondary Endpoints

Secondary endpoints for QUICK-I are shown in table 3. Secondary endpoints will be analyzed for responders and non-responders to treatment, as defined in Table 4.

**Table 3:** Secondary endpoints for QUICK-I

Secondary Endpoints QUICK-I
1. The proportion of patients in whom cardiovascular disease (CVD) risk factor treatment is successful (see definitions) after 1 year of follow up
2. The incidence of target organ damage* and established CVD during the study period
3. The incidence of all cause mortality during the study period
4. Change in the score on the 12-Item Short Form Health Survey quality of life questionnaire at 1 year of follow up compared to baseline
5. Change in the score on the 8-item Morisky Medication Adherence Scale measured with 3 month intervals during one year of follow up
6. Incidence of side effects of prescribed drugs for CVD prevention, during the follow up period

*Target organ damage: presence of micro/macroalbuminuria or ECG based left ventricular hypertrophy

**Table 4:** Definitions of significant improvement per risk factor

*Risk factor*	*Significant improvement*
Blood pressure	Blood pressure at target level:
	Patients without diabetes or established cardiovascular disease (CVD): < 140 mmHg systolic and < 90 mmHg diastolic.
	Patients with diabetes, renal disease or establish CVD: < 130 mm Hg systolic and 80 mmHg diastolic.
	For those who do not reach target levels: blood pressure decrease of > 10% systolic or diastolic

Dyslipidemia	Lipid profile at target level:
	Primary prevention: total cholesterol (TC) < 5.0 mmol/L and low density lipoprotein (LDL) cholesterol < 3.0 mmol/l.
	Secondary prevention: TC < 4.0 mmol/L and LDL cholesterol < 2.5 mmol/l.
	For those who do not reach target levels: reduction in total cholesterol ≥ 25% or LDL ≥ 30% (whichever is greater).

Diabetes	Fasting plasma glucose < 7.0 mmol/l
	Random plasma glucose of < 12 mmol/l

Abdominal Obesity	Reduction of waist circumference to ≤ 102 cm (M), ≤ 88 cm (F)

Left Ventricular Hypertrophy (LVH)	Regression of LVH (electrocardiogram based) according to Sokolov-Lyon criteria [44]

Microalbuminuria	Disappearance of microalbuminuria (< 30 mg/ml in spot morning urine)

Proteinuria	Disappearance of proteinuria (negative quantitative dipstick)

Renal impairment	Improvement in creatinine clearance to a new estimated glomerular filtration rate class [45] after treatment.

Smoking	Quit smoking

### Definitions of clinical outcomes

A patient is considered to be *successfully treated *if there is a significant improvement in any of the risk factors that were present before the start of the prevention program. This implies that each patient reaches an individual endpoint. The definitions of significant improvement are shown in table 4.

### Sample size

The main objective of this study is to describe the cardiovascular care performance of the clinic using quality indicators. Sample size calculations for quality indicator scoring are difficult because the number of cases used to score an indicator depends on the prevalence rate of each condition of interest in the clinic where scoring takes place. Therefore, composite scores, based on pooled indicators of one disease category, are considered better indicators than individual indicator scores [29]. In a study that assessed the feasibility of quality indicator assessment for multiple conditions in general practice, sample sizes of 7-12 patients for each of the conditions were required to achieve 90% confidence intervals of +/- 10 points on estimated pass rates (the number of cases for which the indicator was passed) for composite scores per disease category [29].

The secondary outcome of this project is the proportion of subjects that are successfully treated. In a community based survey in Mozambique, 40% of the participants on treatment for hypertension were found to have a blood pressure at target level [30]. Assuming 30% of patients will be treated successfully, 300 patients will provide sufficiently narrow confidence limits around the estimate (95% CI: 24.9% -35.5%). The study sample size of 300 patients is therefore based on the secondary outcome and will also allow scoring of quality indicators (primary outcome).

A subgroup analysis will be performed on patients who are newly diagnosed or who have not received treatment for at least 1 year ("new" patients), and patients who were identified and put on treatment prior to inclusion into the QUICK-I study.

We aim to enrol 150 new and 150 patients already on treatment (20% success: 95% CI: 13.9% -27.3%; 30% success: 95% CI 22.8%-38.0) within 6 months. Evaluation of the feasibility to reach the sample size and the distribution across the two subgroups, will take place 3 months after the start of inclusion. A minimum sample size of 100 patients in each of the subgroups will be required.

### Statistical Methods

#### Primary outcome

The primary outcome is a descriptive analysis of the quality scores using the NHS QOF indicators, adjusted to the local context, after initiation of standardized CVD management. Only disease domains relevant to CVD will be used (primary prevention, hypertension, diabetes, heart failure, stroke, chronic kidney disease, obesity and smoking). Scoring methods are summarized in Additional file 2. The score will be used to assess the feasibility of CVD prevention care according to the WHO/ISH CVD risk management guidelines [15-17] using the scores of individual NHS QOF indicators. For example, the guideline to perform a CVD risk assessment in all patients with hypertension will be assessed on the basis of the scores of indicator primary prevention 1: "patient newly diagnosed with hypertension had a face to face cardiovascular risk assessment at the outset of diagnosis using an agreed risk assessment tool." The NHS QOF indicators are used in primary health care centres in the UK. The score of the study clinic will also be compared with scores of primary health care practices in the UK. Designated patient files for CVD prevention care were introduced in the clinic prior to the start of the study. These files serve as the source for scoring of quality indicators of patient care provided for the study population included in QUICK- I.

#### Costs and cost-effectiveness analysis

Descriptive statistics will be applied to calculate costs of the CVD prevention program for the clinic, the patients and the insurance company. Estimation of the impact of CVD prevention care on total medical pay out of the insurance company will be done using a mathematical model. Prevalence data of patients at risk for CVD in the insured population will be used as input in this model. Scenario analysis will then be performed to estimate the cost impact for the insurer of different treatment thresholds (e.g. low versus high risk) and with different benefit packages.

Cost effectiveness of standard CVD prevention care in this low resource setting will be estimated using a Markov model with varying probabilities of disease events and mortality to assess the benefits, risks, and costs of the treatment regimes for low, moderate and high CVD risk. Since the analysis will use at least a 10-year time horizon, the long-term effectiveness and cost-effectiveness will be based not only on the QUICK-1 cohort study data, but also on Framingham risk functions (using baseline and follow-up data), relative risk reductions expected from treatment, background mortality rates, and costs of CVD events. Outcomes in the analyses will be measured in QALYs gained and net health-care costs. Multivariate sensitivity analysis will be performed for all variables with a pre-specified range of uncertainty with Monte Carlo simulation.

#### Secondary outcomes

The proportion of patients in whom treatment is successful at 1 year of follow up will be determined for the entire cohort. Individuals who achieved the target will be compared with those who did not and analyzed for explanatory variables using multivariate logistic regression analyses. The proportion of patients who reached treatment target goals will also be determined for each risk factor separately and analyzed using multivariate logistic regression, if sample size allows. The prevalence and incidence of TOD, CVD and mortality with confidence intervals will be determined. Multivariate analyses will be used to adjust for known or suspected confounding variables. Descriptive statistics will be applied for quality of life, side effects of medication and adherence to therapy. Individuals who are adherent to therapy will be compared with non-adherent individuals and analyzed for explanatory variables using multivariate logistic regression analyses.

## Ethical Approval

The QUICK studies will be performed following Good Clinical Practice guidelines and complying with the Declaration of Helsinki principles. Ethical clearance has been obtained from the institutional review board of the University of Ilorin Teaching Hospital (Ref: UITH/CAT/189/13/13, dated 30^th ^March, 2010), Kwara State, Nigeria.

## Start Date & Time Lines

Patient inclusion started in June 2010 and the last patient is expected to complete follow up in December 2011.

## Discussion

Guidelines for CVD care in LMIC have been developed [15-17] but little evidence-based information is available on strategies for effective and successful implementation of such care in settings with low resources. Community health insurance programs have been put forward as a means to improve patient access to healthcare but operational difficulties still hamper the successful development of community health insurance schemes [31]. The context of the insurance program makes this project unique. By removing the barrier of patient out of pocket expenditures through an insurance program, the project can evaluate health effects of the interventions in the program and identify limiting factors for implementation of CVD prevention care. Results of such operational research can be used in future program designs. This project could serve as a model for policy makers and professionals who aim to implement CVD prevention programs in settings with limited resources.

The feasibility of hypertension and diabetes treatment in primary health care in SSA has been assessed in a limited number of studies [32-35]. These studies demonstrate improvements in blood pressure, glucose or HbA1c levels using simple treatment protocols (usually applied by dedicated study nurses). We have chosen for quality indicators as the primary outcome instead of risk factor control. This will give a better insight in which components of CVD care are feasible and which are not when implementing CVD prevention care according to international guidelines, using the available resources, including human resources. However, risk factor reduction after implementation of the CVD program will be described as a secondary outcome.

The NHS QOF indicators are developed in the United Kingdom. There is no CVD prevention quality indicator set developed specifically for low resource settings. WHO/ISH has developed guidelines for the prevention of CVD in LMIC but there are no tools available to measure adherence to these guidelines. In addition, experts disagree which quality indicators are relevant to measure quality of care for primary prevention of CVD [36]. We have chosen the NHS QOF indicators because they have been developed to assess the quality of care in primary health care settings and because the indicators are based on international guidelines. We will describe the results in the context of international CVD prevention guidelines such as the WHO/ISH guidelines [15-17]. Some of the QOF NHS indicators refer to services that are not available in a rural low income setting. We tried to adapt these indicators to the local context wherever possible (for example by replacing the need for an HbA1c test in diabetic patients with a blood glucose test). Other indicators that refer to unavailable services (such as influenza vaccinations for diabetic patients) will fall under the exception reporting criteria. More information on the scoring systems and exception reporting can be found in Additional file 2 and at the QOF NHS website [28].

Previous studies in SSA have focussed on either hypertension or diabetes care [32-35]. We have chosen to include all patients who are at high risk to develop CVD, independent of whether they have hypertension, diabetes or other CVD risk factors. This heterogeneity makes our study population representative of the population which doctors in SSA encounter in their clinics. This approach is in line with WHO recommendations to focus on total CVD risk instead of a single risk factor approach [16].

The financial aspects of delivering CVD care for patients, clinics and third parties (such as insurers) were not addressed in recently published studies [32-35]. However, the cost of care is a major determinant of long term sustainability of CVD prevention programs in SSA. Therefore, the cost of the CVD prevention program is a primary outcome in this study.

Our study population is enrolled in the HCHP program so the study describes the feasibility of CVD prevention care in the context of this insurance program. As the majority of the population in Kwara State is eligible for this insurance program and enrolment rates are high, the results of our study will be applicable to the Kwara population in general. Currently, the insurance program is operational in 2 out of the 3 senatorial districts of Kwara State, expansion to the third area is in preparation. In addition, HIF started a similar program in Tanzania, and a program in Kenya is in preparation. There is increasing advocacy for community-based health insurance schemes as part of a broader solution to health care financing problems in low-income countries [37]. Recently, more community based health insurance schemes have been planned or implemented in SSA [38,39]. Several outcomes of our study, such as the costs of CVD prevention for the clinic, incidence of TOD and travel costs for patients will be relevant for other settings, regardless of whether or not health insurance is available there.

In conclusion, the results of QUICK-I can be used to develop implementation strategies for CVD prevention programs in settings with limited resources. The context of the insurance program will provide insight in the opportunities community health insurance may offer to attain sustainable chronic disease management programs in low resource settings.

## Abbreviations

CVD: Cardiovascular Disease; ECG: Electrocardiogram; HCHP: Hygeia Community Health Plan; HIF: Health Insurance Fund; LMIC: Low and Middle Income Countries; LVH: Left Ventricular Hypertrophy; NHS QOF: National Health Service Quality and Outcome Framework; OOH: Ogo Oluwa Hospital; QALY: Quality Adjusted Life Year; QUICK: Quality Improvement Cardiovascular care Kwara; SF-12: 12-Item Short Form Health Survey; SSA: sub-Saharan Africa; TOD: Target Organ Damage; WHO/ISH: World Health Organization/International Society of Hypertension.

## Competing interests

The authors declare that they have no competing interests.

## Authors' contributions

MH, LB, FW, CS and JL developed the original idea for the study. The study design was further developed by MH, LB, FW, WR, AE, JL, AV and CS. The following authors have contributed to the design and planning of the quality improvement program described in this study: MH, LB, OAB, AOO, NB, SA, PA, KA, TA, AV and CS (training of the participant doctors, nurses and administrative staff, support material, etc). MH and FW developed the analytical plan and will carry out the analysis. The analytical plan for all costs related outcomes was designed by MH and WR. All authors have read, corrected draft versions and approved the final version of the manuscript.

## Pre-publication history

The pre-publication history for this paper can be accessed here:

http://www.biomedcentral.com/1471-2458/11/186/prepub

## Supplementary Material

Additional file 1 **Appendix 1: Treatment flowcharts**. Click here for file

Additional file 2 **Appendix 2: Quality indicators**. Click here for file
