# Supplementary material for: Cardiovascular disease prevention in rural Nigeria in the context of a community based health insurance scheme: QUality Improvement Cardiovascular care Kwara-I (QUICK-I)
Source: BMC Public Health. 2011 Mar 25;11:186. doi: 10.1186/1471-2458-11-186 (PMC3073902; doi:10.1186/1471-2458-11-186)
Supplement: Additional file 1 — Appendix 1: Treatment flowcharts. [file 1471-2458-11-186-S1.PDF]

Appendix 1: flowcharts for cardiovascular disease prevention

The risk assessment and treatment flowcharts for cardiovascular disease (CVD) prevention care used in this project are based on international guidelines of the Joint National Committee on Prevention, Detection, Evaluation, and Treatment of High Blood Pressure [1], the European Society of Hypertension/European Society of Cardiology [2] and the World Health Organization/International Society of Hypertension [3-5] , adapted to the local context as explained below. The flowchart for diabetes care is based on guidelines by WHO, the American Diabetes Association [3, 5-7] and a flowchart designed by Gill et al. for diabetes care in rural South Africa [7]. Drug choices indicated in the flowcharts are based on local availability and costs.

Women in fertile period

In pregnant women, the first choice of treatment is alpha methyldopa and labetolol combination therapy [2]. Angiotensin Converting Enzyme Inhibitors (ACEI) have shown to be teratogenic and Angiotensin II Receptor Blockers (ARBs) have not been proven to be safe during pregnancy. A large proportion of the women in the setting of this project do not take birth control measures, therefore ACEI (and ARBs but these are not first line therapy in this setting) are contra indicated for all women in the fertile age (18- 45years) unless they take birth control measures. The first choice therapy for these women is a calcium channel blocker, if at high risk for CVD combined with methyldopa. If blood pressure is not controlled, hydralazine (labetolol is not available in this setting) is recommended as additional therapy. Pregnant women are excluded from QUICK-I, they will receive standard care according to guidelines.

Antiplatelet therapy

Low dose of antiplatelet therapy is recommended for primary prevention in high risk groups provided that blood pressure is controlled. WHO guidelines state that the benefit of antiplatelet therapy could be lower in populations where hemorrhagic stroke is more prevalent than ischemic heart disease [3]. It is expected that the study population consist predominantly of patients with hypertension. The risk of adding aspirin for all high risk patients is considered too high because of the risk of hemorrhagic stroke. It will be left to the doctors' judgment if treatment with antiplatelet therapy is beneficial in individual cases. For secondary stroke prevention, WHO recommends low dose aspirin in presumed ischemic stroke. Hemorrhagic stroke is a contraindication for antiplatelet therapy. In the setting of this project, differentiation between hemorrhagic and ischemic stroke is not possible. Antiplatelet therapy is therefore not standard treatment for secondary stroke prevention. Again, the decision to start antiplatelet therapy in individual cases will be left to the doctors judgment [8].

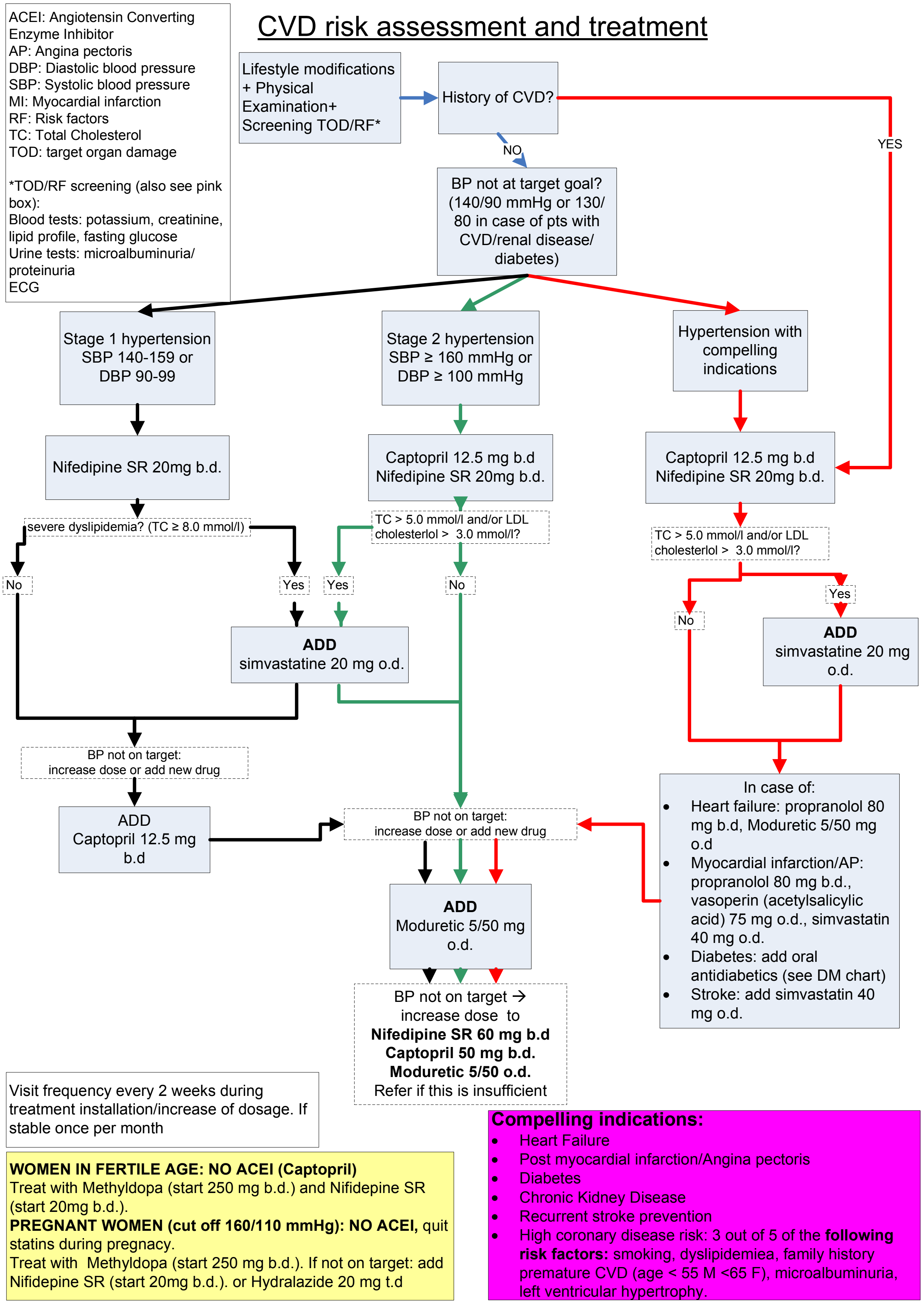

Figure 1: CVD risk assessment and treatment. Choices of drugs are based on international guidelines [1-5] and local availability.

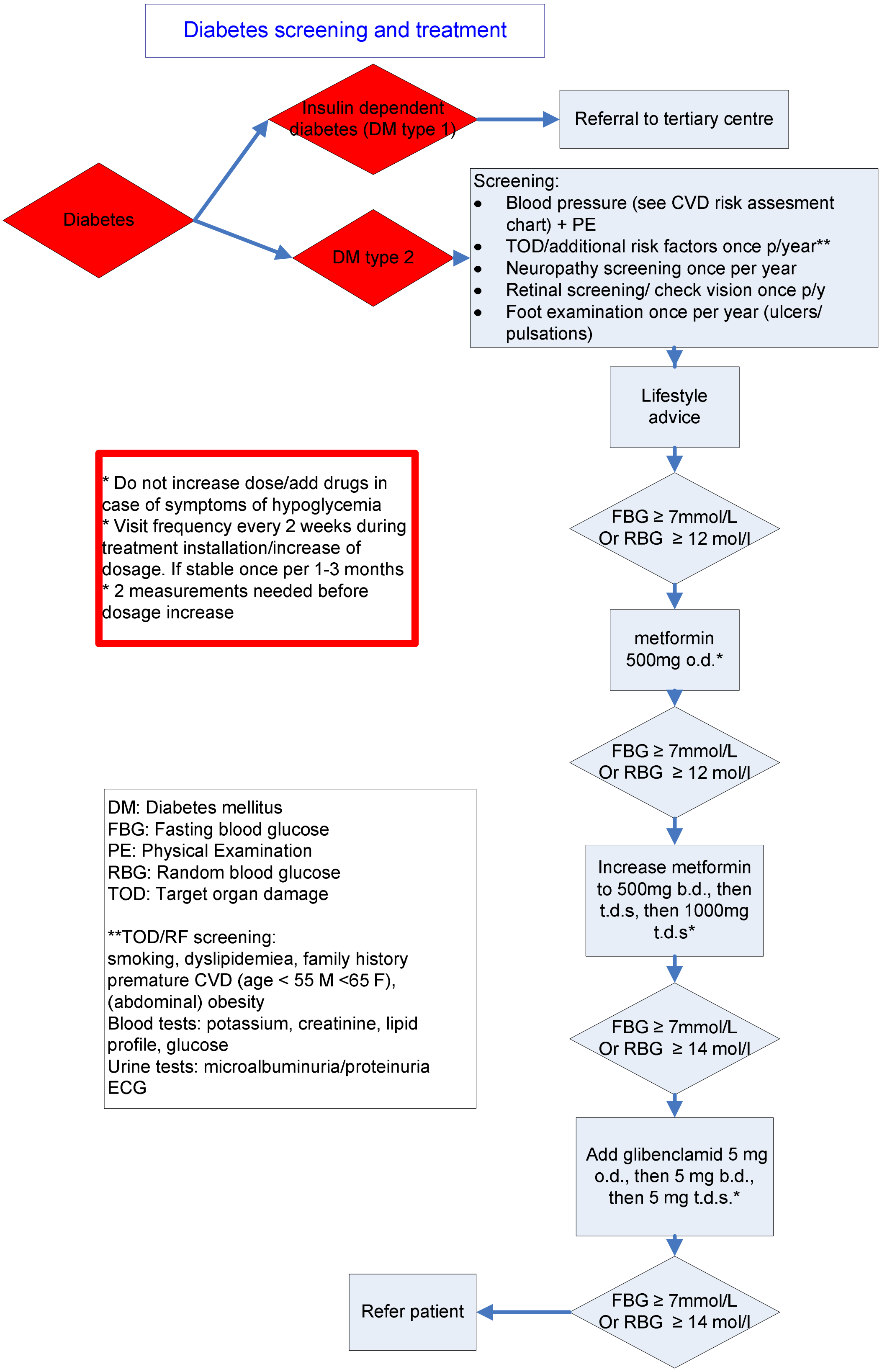

Figure 2: Diabetes treatment. Choices of drugs are based on international guidelines [3, 5-7] and local availability.

References

1. Chobanian AV, Bakris GL, Black HR, Cushman WC, Green LA, Izzo JL, Jr., Jones DW, Materson BJ, Oparil S, Wright JT, Jr., Roccella EJ: **The Seventh Report of the Joint National Committee on Prevention, Detection, Evaluation, and Treatment of High Blood Pressure: the JNC 7 report.** *Jama* 2003, **289:**2560-2572.
2. Mancia G, De Backer G, Dominiczak A, Cifkova R, Fagard R, Germano G, Grassi G, Heagerty AM, Kjeldsen SE, Laurent S, Narkiewicz K, Ruilope L, Rynkiewicz A, Schmieder RE, Boudier HA, Zanchetti A, Vahanian A, Camm J, De Caterina R, Dean V, Dickstein K, Filippatos G, Funck-Brentano C, Hellemans I, Kristensen SD, McGregor K, Sechtem U, Silber S, Tendera M, Widimsky P, Zamorano JL, Erdine S, Kiowski W, Agabiti-Rosei E, Ambrosioni E, Lindholm LH, Viigimaa M, Adamopoulos S, Agabiti-Rosei E, Ambrosioni E, Bertomeu V, Clement D, Erdine S, Farsang C, Gaita D, Lip G, Mallion JM, Manolis AJ, Nilsson PM, O'Brien E, Ponikowski P, Redon J, Ruschitzka F, Tamargo J, van Zwieten P, Waeber B, Williams B: **2007 Guidelines for the Management of Arterial Hypertension: The Task Force for the Management of Arterial Hypertension of the European Society of Hypertension (ESH) and of the European Society of Cardiology (ESC).** *Journal of hypertension* 2007, **25:**1105-1187.
3. World Health Organization: *Prevention of Cardiovascular Disease. Guidelines for assessment and management of cardiovascular risk.* Geneva: WHO Press; 2007.
4. Mendis S, Lindholm LH, Mancia G, Whitworth J, Alderman M, Lim S, Heagerty T: **World Health Organization (WHO) and International Society of Hypertension (ISH) risk prediction charts: assessment of cardiovascular risk for prevention and control of cardiovascular disease in low and middle-income countries.** *Journal of hypertension* 2007, **25:**1578-1582.
5. World Health Organization: *Prevention of cardiovascular disease : pocket guidelines for assessment and management of cardiovascular risk : (WHO/ISH cardiovascular risk prediction charts for the African Region).* Geneva: WHO Press; 2007.
6. **Standards of medical care in diabetes--2009.** *Diabetes Care* 2009, **32 Suppl 1:**S13-61.
7. Gill GV, Price C, Shandu D, Dedicoat M, Wilkinson D: **An effective system of nurse-led diabetes care in rural Africa.** *Diabet Med* 2008, **25:**606-611.
8. Baigent C, Blackwell L, Collins R, Emberson J, Godwin J, Peto R, Buring J, Hennekens C, Kearney P, Meade T, Patrono C, Roncaglioni MC, Zanchetti A: **Aspirin in the primary and secondary prevention of vascular disease: collaborative meta-analysis of individual participant data from randomised trials.** *Lancet* 2009, **373:**1849-1860.
