# Supplementary material for: Cardiovascular disease prevention in rural Nigeria in the context of a community based health insurance scheme: QUality Improvement Cardiovascular care Kwara-I (QUICK-I)
Source: BMC Public Health. 2011 Mar 25;11:186. doi: 10.1186/1471-2458-11-186 (PMC3073902; doi:10.1186/1471-2458-11-186)
Supplement: Additional file 2 — Appendix 2: Quality indicators. [file 1471-2458-11-186-S2.PDF]

Appendix 2: Adjusted National Health Service Quality and Outcome Framework quality indicators

The United Kingdom National Health Service Quality and Outcome Framework (NHS QOF) contains groups of indicators, against which a primary care practice can obtain points according to their level of achievement. The British National Institute of Clinical Excellence (NICE) advises on relevant, evidence based quality indicators, which includes a number of domains such as coronary heart disease and hypertension. In this project, only indicators relevant for CVD (prevention) will be used. The number of cases in the denominator to calculate the score stage will be the number of patients included in QUICK- I who are diagnosed with the condition of interest. Some indicators are related to services that are not available in this setting; these indicators will fall under exception reporting criteria and will not be taken into account when scoring the quality of care. More information on the scoring systems can be found at the NHS QOF website [1].

The indicators have been adapted to the local context and are listed in the tables below.

Table 1: primary prevention indicators

Number	Indicator	Points	Score* stages
PP1	The percentage of patients with a new diagnosis of hypertension (excluding those with pre-existing coronary heart disease (CHD), diabetes, stroke and/or transient ischemic attack (TIA)) recorded in the last year who had a face to face cardiovascular risk assessment at the outset of diagnosis (within 3 months of the initial diagnosis) using an agreed risk assessment tool.	8	40-70%
PP2	The percentage of patients diagnosed with hypertension diagnosed after 1 April 2009 who are given lifestyle advice in the last 15 months for: increasing physical activity, smoking cessation, safe alcohol consumption and healthy diet.	5	40-70%

*see reference 1

Table 2: Hypertension indicators

Number	Indicator	Points	Score* stages
BP1	The practice can produce a register of patients with established hypertension.	6	
BP4	The percentage of patients with hypertension in whom there is a record of the blood pressure in the previous 9 months.	18	40-90%
BP5	The percentage of patients with hypertension in whom the last blood pressure (measured in the previous 9 months) is 150/90 or less.	57	40-70%

*see reference 1

Table 3: Heart failure indicators

Number	Indicator	Points	Score* stages
HF1	The practice can produce a register of patients with heart failure.	4	
HF2	The percentage of patients with a diagnosis of heart failure (diagnosed after 1 April 2006) which has been confirmed by an echocardiogram or by specialist assessment within 12 months of diagnosis in the clinic	6	40-90%
HF3	The percentage of patients with a current diagnosis of heart failure due to Left Ventricular Dysfunction (LVD) who have been prescribed an angiotensin converting enzyme inhibitor (ACE) inhibitor or an angiotensin receptor blocker (ARB) in the previous 6 months, who can tolerate therapy and for whom there is no contraindication.	10	40-80%
HF4	The percentage of patients with a current diagnosis of heart failure due to LVD who have been prescribed an ACE inhibitor or an ARB in the previous 6 months, who are additionally treated with a beta blocker, or recorded as intolerant to or having a contraindication to beta-blockers.	9	40-60%

*see reference 1

Table 4: Stroke indicators

Number	Indicator	Points	Score* stages
Stroke1	The practice can produce a register of patients with stroke or transient ischemic attack (TIA).	2	
Stroke13	The percentage of new patients with a stroke or TIA who have been referred for further investigation.	2	40-80%
Stroke5	The percentage of patients with TIA or stroke whose notes have a record of blood pressure in the preceding 15 months.	2	40-90%
Stroke6	The percentage of patients with a history of TIA or stroke in whom the last blood pressure reading (measured in the previous 15 months) is 150/90 or less.	5	40-70%
Stroke7	The percentage of patients with TIA or stroke who have a record of total cholesterol in the past 15 months.	2	40-90%
Stroke8	The percentage of patients with TIA or stroke whose last measured total cholesterol (measured in the previous 15 months) is 5 mmol/L or less.	5	40-60%
Stroke12	The percentage of patients with a stroke shown to be non-haemorrhagic, or a history of TIA, who have a record that an anti-platelet agent (aspirin, clopidogrel, dipyridamole or a combination), or oral anti-coagulant is being taken (unless a contraindication or side-effects are recorded).	4	40-90%
Stroke10	The percentage of patients with TIA or stroke who have a record of influenza immunization in the preceding 1 September to 31 March.	2	40-85%

*see reference 1

Table 5: Diabetes indicators

Number	Indicator	Points	Score* stages
DM19	The practice can produce a register of all patients aged 17 years and over with diabetes mellitus.	6	40-90%
DM2	The percentage of patients with diabetes whose notes record Body Mass Index (BMI) in the previous 15 months.	3	40-90%
DM5	The percentage of patients with diabetes who have a record of HbA1c or blood glucose in the previous 15 months.	3	40-90%
DM23	The percentage of patients with diabetes in whom the last HbA1c is 7 or less in the previous 15 months	17	40-50%
DM24	The percentage of patients with diabetes in whom the last HbA1c is 8 or less, or fasting blood glucose is 7 mmol/l or less, or random blood glucose is 14 mmol/l or less in the previous 15 months.	8	40-70%
DM25	The percentage of patients with diabetes in whom the last HbA1c is 9 or less in the previous 15 months.	10	40-90%
DM21	The percentage of patients with diabetes who have a record of retinal screening/vision check in the previous 15 months.	5	40-90%
DM9	The percentage of patients with diabetes with a record of the presence or absence of peripheral pulses in the previous 15 months.	3	40-90%
DM10	The percentage of patients with diabetes with a record of neuropathy testing in the previous 15 months.	3	40-90%
DM11	The percentage of patients with diabetes who have a record of blood pressure in the previous 15 months.	3	40-90%
DM12	The percentage of patients with diabetes in whom the last blood pressure is 145/85 or less, measured in the last 15 months.	18	40-60%
DM13	The percentage of patients with diabetes who have a record of micro-albuminuria testing in the previous 15 months (exception reporting for patients with proteinuria).	3	40-90%
DM22	The percentage of patients with diabetes who have a record of estimated glomerular filtration rate (eGFR) or serum creatinine testing in the previous 15 months.	3	40-90%
DM15	The percentage of patients with diabetes with a diagnosis of proteinuria or micro-albuminuria who are treated with an angiotensin converting enzyme inhibitor (ACE) inhibitors (or A2 antagonists) in the last 6 months).	3	40-80%
DM16	The percentage of patients with diabetes who have a record of total cholesterol in the previous 15 months	3	40-90%
DM17	The percentage of patients with diabetes whose last measured total cholesterol within the previous 15 months is 5 mmol/l or less.	6	40-70%
DM18	The percentage of patients with diabetes who have a record of influenza immunization in the preceding 1 September to 31 March.	3	40-85%

*see reference 1

Table 6: Chronic Kidney Disease indicators

Number	Indicator	Points	Score* stages
CKD1	The practice can produce a register of patients aged 18 years and over with chronic kidney disease (CKD) (US National Kidney Foundation: Stage 3 to 5 CKD).	6	
CKD2	The percentage of patients with CKD whose notes have a record of blood pressure in the previous 15 months.	6	40-90%
CKD3	The percentage of patients with CKD in whom the last blood pressure reading, measured in the previous 15 months, is 140/85 or less.	11	40-70%
CKD5	The percentage of patients with CKD with hypertension and proteinuria who are prescribed an angiotensin converting enzyme inhibitor (ACE-I) or angiotensin receptor blocker (ARB) (unless a contraindication or side effects are recorded), in the last 6 months.	9	40-80%
CKD6	The percentage of patients with CKD whose notes have a record of a urine albumin: creatinine ratio (ACR) or protein: creatinine ratio (PCR) test recorded in the previous 15 months.	6	40-80%

*see reference 1

Table 7: Obesity indicators

Number	Indicator	Points	Score* stages
OB1	The practice can produce a register of patients aged 18 and over with a Body Mass Index (BMI) greater than or equal to 30 in the previous 15 months.	8	

*see reference 1

Table 8: Smoking indicators

Number	Indicator	Points	Score* stages
Smoking 3	The percentage of patients with any or any combination of the following conditions: coronary heart disease (CHD), stroke or transient ischemic attack (TIA), hypertension, diabetes, Chronic Obstructive Pulmonary Disease (COPD), chronic kidney disease (CKD), asthma, schizophrenia, bipolar affective disorder or other psychoses whose notes record smoking status in the previous 15 months.	30	40-90%
Smoking 4	The percentage of patients with any or any combination of the following conditions: CHD, stroke or TIA, hypertension, diabetes, COPD, CKD, asthma, schizophrenia, bipolar disorder or other psychoses who smoke whose notes contain a record that smoking cessation advice or referral to a specialist service, where available, has been offered within the previous 15 months.	30	40-90%

*see reference 1

References

1. **Quality and Outcomes Framework guidance for GMS contract 2009/10** [http://www.nhsemployers.org/Aboutus/Publications/Documents/QOF_Guidance_2009_final.pdf]
